# Assessment of right ventricular sympathetic dysfunction in patients with arrhythmogenic right ventricular cardiomyopathy: An ^123^I-metaiodobenzylguanidine SPECT/CT study

**DOI:** 10.1007/s12350-018-01545-3

**Published:** 2018-12-17

**Authors:** Andrei Todica, Johannes Siebermair, Julia Schiller, Mathias J. Zacherl, Wolfgang P. Fendler, Steffen Massberg, Peter Bartenstein, Clemens C. Cyran, Stefan Kääb, Marcus Hacker, Reza Wakili, Sebastian Lehner

**Affiliations:** 1 Department of Nuclear Medicine, University of Munich, Munich, Germany; 2 Department of Cardiology and Vascular Medicine, West-German Heart and Vascular Center Essen, University Duisburg-Essen, Essen, Germany; 3 Department of Cardiology, University of Munich, Munich, Germany; 4 Department of Clinical Radiology, University of Munich, Munich, Germany; 5 Division of Nuclear Medicine, Department of Biomedical Imaging and Image-Guided Therapy, Medical University Vienna, Währinger Gürtel 18-20, 1090 Vienna, Austria; 6 Ambulatory Healthcare Center Dr. Neumaier & Colleagues, Radiology, Nuclear Medicine, Radiation Therapy, Regensburg, Germany

**Keywords:** ARVC, MIBG, SPECT, IVF, Quantification, Sympathetic innervation

## Abstract

**Purpose:**

The purpose of the study was to evaluate a novel approach for the quantification of right ventricular sympathetic dysfunction in patients diagnosed with ARVC/D through state-of-the-art functional SPECT/CT hybrid imaging.

**Methods:**

Sympathetic innervation of the heart was assessed using ^123^I-MIBG-SPECT/CT in 17 patients diagnosed with ARVC according to the modified task force criteria, and in 10 patients diagnosed with idiopathic ventricular fibrillation (IVF). The ^123^I-MIBG-uptake in the left (LV) and right ventricle (RV) was evaluated separately based on anatomic information derived from the CT scan, and compared to the uptake in the mediastinum (M).

**Results:**

There was a significant difference in the LV/M ratio between the ARVC/D and the IVF groups (3.2 ± 0.5 vs. 3.9 ± 0.8, *P* = 0.014), with a cut-off value of 3.41 (77% sensitivity, 80% specificity, AUC 0.78). There was a highly significant difference in the mean RV/M ratios between both groups (1.6 ± 0.3 vs. 2.0 ± 0.2, *P* = 0.001), with optimal cut-off for discrimination at 1.86 (88% sensitivity, 90% specificity, AUC 0.93).

**Conclusion:**

Employing state-of-the-art functional SPECT/CT hybrid imaging, we could reliably assess and quantify right and left ventricular sympathetic innervation. The RV/M ratio was significantly lower in patients diagnosed with ARVC/D and provided sensitive and specific discrimination between patients with ARVC/D and IVF patients.

**Electronic supplementary material:**

The online version of this article (10.1007/s12350-018-01545-3) contains supplementary material, which is available to authorized users.

## Introduction

Arrhythmogenic right ventricular cardiomyopathy/dysplasia (ARVC/D) is a mainly inherited disease of heart muscle, which is characterized by a progressive replacement of contractile heart muscle with fibrotic and fat tissues (fibrofatty degeneration), primarily in the right ventricle (RV).^1^ The progression of RV muscle disease and the involvement of the left ventricle (LV) can ultimately lead to heart failure.^2^ Arrhythmia arising from undiagnosed ARVC is notoriously a cause of sudden cardiac death in young people and athletes.^3^ The prevalence of ARVC/D in the general population may be as high as 0.05%, with a threefold higher occurrence in men than in women. Over 50% of ARVC/D cases have a familial background, with the usual mode of inheritance as an autosomal-dominant trait with variable expression and incomplete penetrance, such that not everyone bearing an implicated genetic mutation will develop the disease.^4^

The diagnosis of ARVC/D is especially hindered at early stages due to the absence or non-specific nature of clinical findings, and due to its multifactorial genesis, in conjunction with the anatomic complexity of RV function and failure. Therefore, there has been no single diagnostic gold standard for detection of ARVC/D, but rather a reliance on combining results from several diagnostic examinations. Currently the diagnosis of ARVC/D is based on the presence of major and minor criteria including ECG re- and depolarization abnormalities, ventricular arrhythmias, RV function and morphology, histopathology, and family history. The diagnosis is established when two major, one major plus two minor, or four minor criteria from different groups are fulfilled, as suggested by the modified Task Force Criteria.^5^

Molecular imaging with single photon computer tomography (SPECT) might provide improved sensitivity and specificity for the diagnosis of ARVC/D. ^123^I-Metaiodobenzylguanidine (^123^I-MIBG) is an analogue of guanethidine, which is an established agent for visualizing the sympathetic innervation of myocardium and other tissues.^6^ In Its uptake in sympathetic fibers is mediated by the plasma membrane noradrenaline transporter, and its long-term retention gives an index of the functional capacity of the sympathetic nervous system.^7^,^8^

Initial cardiac imaging studies revealed abnormal sympathetic innervation patterns in the left ventricle in those patients which was first described by Wichter et al.^9^ The authors therefore hypothesized that the sympathetic innervation of the heart may be involved in the early diagnosis in ARVC/D patients. Further results of initial SPECT studies in ARVC/D patients indicate that reduced ^123^I-MIBG-uptake in the LV myocardium is associated with higher risk of future recurrence of life-threatening ventricular tachyarrhythmia.^10^ A major limitation of the few published ^123^I-MIBG studies investigating ARVC/D patients was the restricted focus on the reduction in LV uptake, without considering changes in the RV uptake. Based on the understanding that the ARVC/D pathology first arises in the right ventricle, we hypothesized that the diagnosis should be better supported by quantitation of right ventricular ^123^I-MIBG uptake (RVU). Therefore, we prospectively evaluated the myocardial sympathetic function using ^123^I-MIBG SPECT/CT in patients diagnosed with ARVC/D and, as a reference, also in patients with idiopathic ventricular fibrillation (IVF).

## Methods

### Study Population

Over the course of 46 months, 27 consecutive patients were investigated at our clinic. Of these, 17 (13 men, 4 women; mean age 49 ± 16 years) met the ARVC/D diagnosis according to the modified task force criteria. The remaining 10 patients (6 men, 4 women; mean age 51 ± 15 years) were diagnosed with IVF, with exclusion of ARVC/D according to the modified task force criteria; this patient group served as our reference cohort for molecular imaging. This study was conducted with the approval of the local ethics committee, with provision of informed consent from all patients.

### Image Acquisition and Data Analysis

SPECT data acquisition was performed in accordance to previous published protocols, with fasting on the day of the scan.^9^–^11^ In brief, after prophylaxis against thyroid uptake with 300 mg perchlorate, a slow intravenous infusion of approximately 350 MBq ^123^I-MIBG was applied over a span of 2 minutes. Planar images of the thorax were acquired at approximately 15 minutes and 4 hours after tracer injection (duration 10 minutes, 128 × 128 matrix, and zoom 1.0). Following completion of the second planar acquisition, a SPECT/CT was acquired using a low energy, high-resolution collimator (90° configuration, 64 × 64 matrix, zoom 1.45, 32 projections, 30 seconds per projection). Ancillary to the previously mentioned protocols an additional low-dose CT (130 keV, 20 mAs) for attenuation correction and morphological co-registration was performed. All scans were performed with a Symbia TruePoint SPECT/CT scanner (Siemens Medical Systems, Erlangen, Germany).

Planar ^123^I-MIBG images were analyzed using the Hermes Hybrid Viewer (Hermes Medical Solutions, Stockholm, Sweden) by manually drawing regions of interest (ROIs), as previously described.^12^ The entire heart ROI was manually drawn around the summation image of the whole heart (right and left ventricles), with particular attention to exclusion of the lungs and the liver. A standardized square ROI (10 mm × 10 mm) was placed upon the mediastinum, with sufficient distance from the lungs and the thyroid gland so as to avoid spill-in. The mean counts per pixel were recorded in the early and delayed planar acquisitions, and entire heart-to-mediastinum ratios were calculated. Furthermore, percentage washout ratios (WOR) were calculated from early and delayed images, as described previously;^6^ this gives an index of the rate of decrease in myocardial counts over time, normalized to the mediastinal ROI.

**Figure 1:**
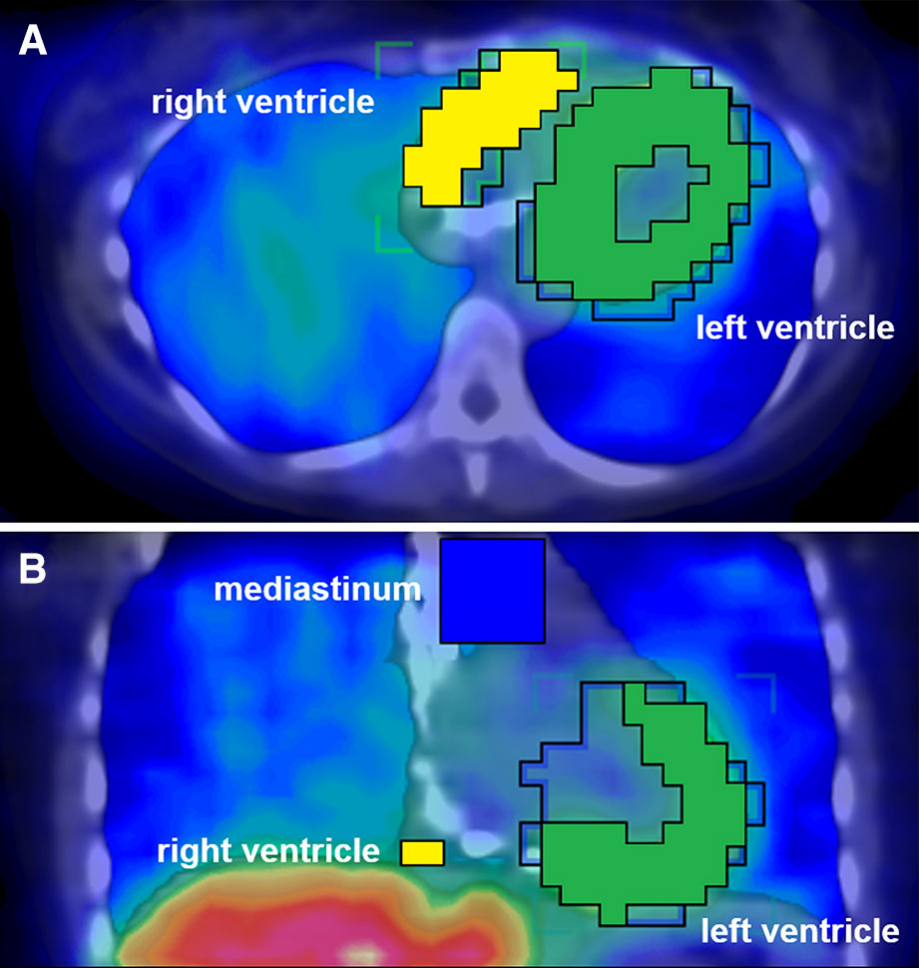
Hybrid SPECT/CT fusion scan. Representative image for VOI definition for the right (yellow) and left (green) ventricle as well as the mediastinum (blue) in transverse (**A**) and coronal (**B**) planes

SPECT/CT images were reconstructed with attenuation and scatter correction (HybridRecon-Cardiology, Hermes Medical Solutions, Stockholm, Sweden), and analyzed using the Inveon Research Workplace (Siemens Medical Solutions, Knoxville, TN). In this analysis, we imported and fused the SPECT and CT images, and manually drew volumes of interest (VOIs) around the left and right ventricle in the CT scan, which were then applied to the SPECT images (see Figure 1). To define the myocardial borders within the VOIs, we applied a threshold of the hottest voxels ranging from 30% to 100% for the RV and 70% to 100% for the LV (for a detailed description of the threshold definition, see Supplemental file). Furthermore, we applied a cubic VOI (33 × 33 × 33 mm) on the upper mediastinum, guided by the morphological information in the CT scan (see Figure 1), and then calculated heart-to-mediastinum ratios for the left and right ventricles.

### Statistical Analysis

All variables are reported as mean ± standard deviation (SD). Variables were tested for normal distribution using the Kolmogorov–Smirnov test. The paired and unpaired Student’s *t*-tests were used where appropriate. For groups without normal distribution, we applied the Wilcoxon signed-rank or the Mann-Whitney-*U* test. Bivariate correlations were calculated using the Spearman or Pearson correlation coefficient. Differences were considered statistically significant at a *P*-value < 0.05. To determine the optimal threshold value, we calculated the area under to the curve of the receiver-operating-characteristics (ROC).

## Results

### Planar Image Analysis

**Figure 2:**
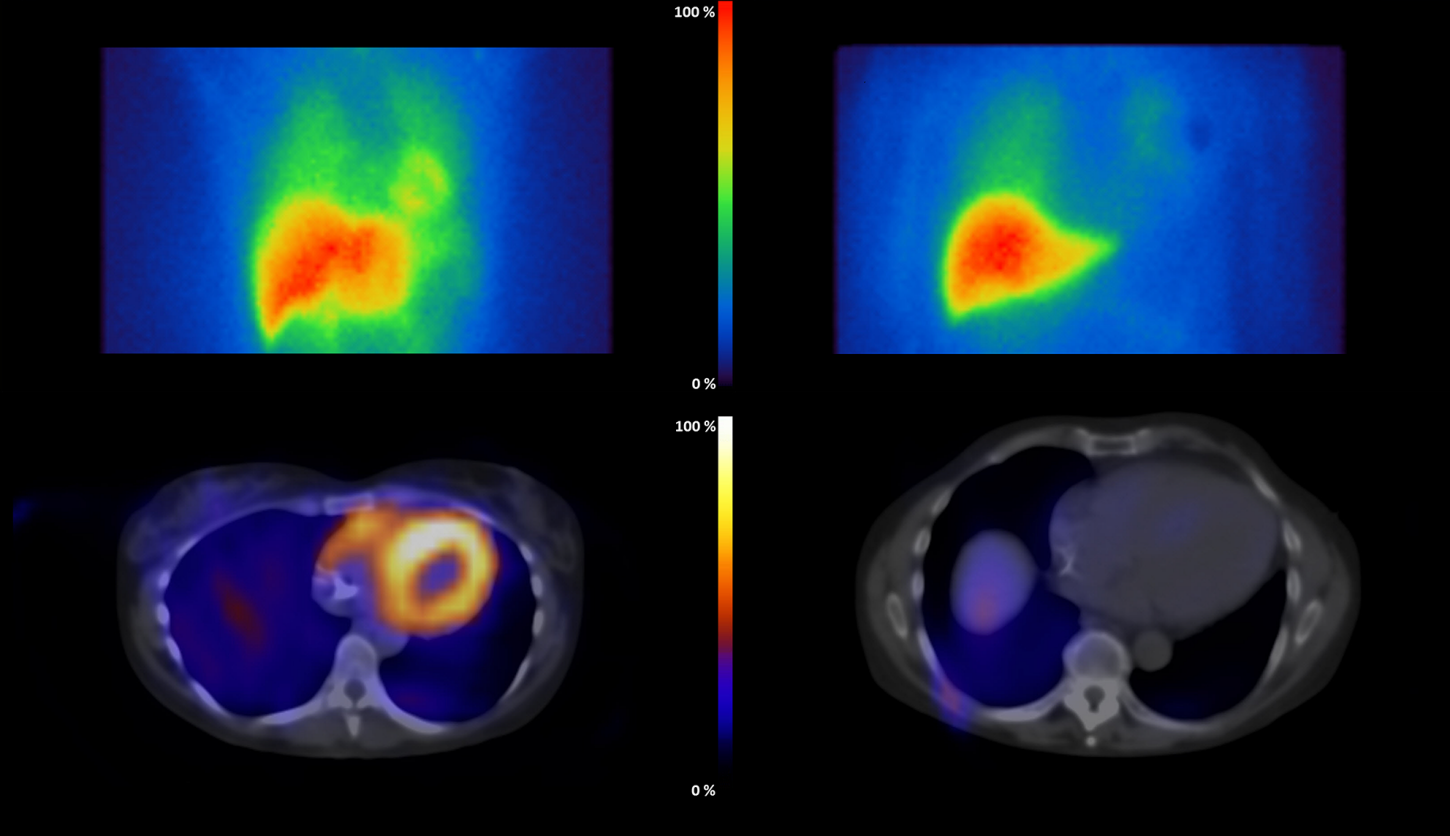
Representative planar and tomographic images of cases with normal (left) and reduced (right) ^123^I-MIBG uptake. Normal (left; IVF group, H/M ratio = 1.87, LV/M ratio = 3.76, RV/M ratio = 2.01) and reduced (right; ARVC/D group, H/M ratio = 1.12, LV/M ratio = 1.79, RV/M ratio = 1.34) uptake as planar (top) and SPECT/CT image (bottom)

The mean planar H/M ratios of the early and delayed images were slightly lower in the ARVC/D group as compared to the IVF reference group, but this difference did not reach statistical significance according to unpaired Student’s *t*-test (early 1.6 ± 0.2 vs. 1.8 ± 0.2, *P* = 0.196; delayed 1.5 ± 0.3 vs. 1.7 ± 0.2, *P* = 0.068); the WOR was non-significantly higher in the ARVC/D group (50% ± 14% vs. 42% ± 8%, *P* = 0.123) (representative images see Figure 2 top).

### SPECT Image Analysis

**Figure 3:**
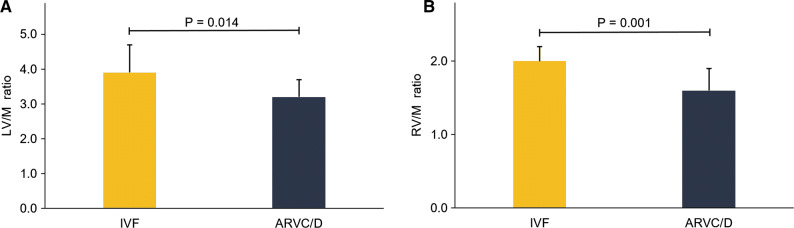
Ventricle-to-mediastinum ratios in the IVF and the ARVC/D group. The LV/M ratio (3.9 ± 0.8 vs. 3.2 ± 0.5) as well as the RV/M ratio (2.0 ± 0.2 vs. 1.6 ± 0.3) was significantly higher in the IVF group as compared to the ARVC/D group. (*ARVC/D*,  arrhythmogenic right ventricular cardiomyopathy / dysplasia; *IVF*, idiopathic ventricular fibrillation; *LV/M ratio*, left ventricle-to-mediastinum ratio; *RV/M ratio*, right ventricle-to-mediastinum ratio)

The LV/M ratio was significantly lower in the ARVC/D group than in the IVF group (3.2 ± 0.5 vs 3.9 ± 0.8 vs., *P* = 0.014; Figure 3A), as was likewise the RV/M ratio (1.6 ± 0.3 vs. 2.0 ± 0.2, *P* = 0.001; Figure 3B) (representative images see Figure 2 bottom).

### Correlation Between Planar Imaging and SPECT/CT

There was a moderate but significant positive correlation between the delayed (4 hours) planar H/M ratio and LV/M ratio (Pearson’s *r* = 0.622, *P* = 0.001) as well as to RV/M ratio (Pearson’s *r* = 0.414, *P* = 0.035).

### ROC Analysis

**Figure 4:**
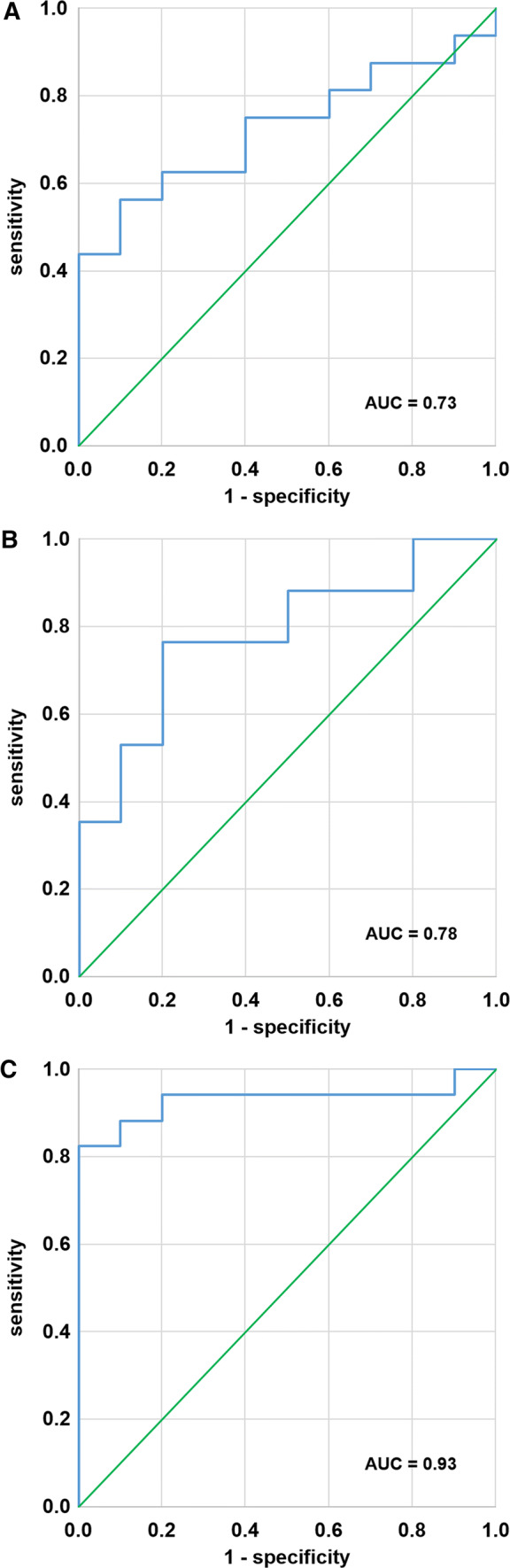
ROC analysis for planar, as well as SPECT/CT based analysis. Optimal cut-off values were 1.66 for the planar H/M ratio (75% sensitivity, 60% specificity; (**A**), 3.41 for the LV/M ratio (77% sensitivity, 80% specificity; (**B**), and 1.86 for the RV/M ratio (88% sensitivity, 90% specificity; (**C**). (*AUC*, area under the curve)

The optimal planar H/M ratio to discriminate between ARVC/D and IVF was 1.66 with 75% sensitivity, 60% specificity, and area under the curve (AUC) of 0.73 (Figure 4A). The ROC-analysis revealed an optimal cut-off value of 3.41 for the LV/M ratio, giving 77% sensitivity, 80% specificity, and an AUC of 0.78 (Figure 4B). For the RV/M ratio, the optimal cut-off value was 1.86, which gave 88% sensitivity, 90% specificity (Figure 4C) and an AUC of 0.93 (see also Table 2 in the supplemental file). An overview over the calculated cut-off values with the corresponding sensitivity and specificity for the H/M ratio, LV/M ratio, and RV/M ratio is presented in Table 3 of the supplemental file.

## Discussion

The objective of this study was to examine a novel hybrid SPECT/CT molecular imaging approach for detecting myocardial sympathetic dysfunction in patients with suspected or diagnosed ARVC/D by analyzing the left and right ventricular uptake separately. To this end, we performed early and delayed planar and delayed SPECT/CT ^123^I-MIBG imaging in a group of 27 patients, 17 of whom proved to have ARVC/D. Using the SPECT/CT fusion images, we were able to obtain separate assessment of the left and right ventricular sympathetic innervation. Whereas planar scintigraphy provided only limited information, the delayed RV/M ratio in SPECT/CT was significantly lower in patients with diagnosed ARVC/D and could distinguish with high sensitivity and specificity between ARVC/D patients and patients with other underlying cardiac heart diseases in our study cohort. To our knowledge, this is the first SPECT/CT study to demonstrate the feasibility of separate left and right ventricular in vivo evaluation of the sympathetic myocardial innervation.

### Planar H/M and Washout Ratio

A number of different ROI-based methods to quantify the myocardial sympathetic innervation in planar images have been published,^13^–^17^ but all different methods showed similar results and correctly predicted a worse prognosis for patients with a low H/M ratio. The patients in our ARVC/D group had a mean H/M ratio of 1.5, slightly below the published cut-off value of 1.6 in the ADMIRE-HF study.^15^ Thus, our slightly lower H/M ratio is in line with earlier findings, since ARVC/D is apparently resulting in partial myocardial sympathectomy, resulting in lower ^123^I-MIBG uptake. The mean H/M ratio in our reference group was 1.7, indicating generally less severe heart disease than in the ARVC/D group. The small numerical difference did not differ significantly between groups, likely because the planar H/M ratio is weighted towards tracer uptake in the left ventricle, which is relatively spared in earlier stages of the disease.

In a previous ^123^I-MIBG study, Ogita et al.^18^ showed that a healthy cohort had a WOR of 9.6% ± 8.5%, whereas those patients with a WOR above 27% had significantly higher mortality than did patients with WOR < 27%. Both of our cohorts showed a WOR much greater than 27%, probably due to the nature of their underlying arrhythmogenic diseases. Nevertheless, the WOR was distinctly higher in our ARVC/D group, pointing towards a more pronounced impairment of the myocardial innervation, as compared to that in the reference group.

### SPECT/CT LV/M and RV/M Ratio

Planar scintigraphy is the established method in the clinical setting to quantify the sympathetic innervation with ^123^I-MIBG. As a 3D modality, SPECT/CT can bring additional valuable information about the distribution of tracer uptake in the heart. In our approach, we used the additional morphological information of the low-dose CT scan to define separate VOIs of the left and right ventricle.

Veen et al. demonstrated a high correlation between planar and left ventricular H/M ratios,^12^ which is in good accordance with our findings. We could demonstrate a systematic underestimation of the cardiac ^123^I-MIBG uptake by planar imaging as compared to SPECT, most likely arising from the somewhat higher mediastinal tracer uptake in planar imaging, also reported by Chen et al.^19^ In an early SPECT study of 48 patients with ARVC/D Wichter et al. showed abnormally reduced ^123^I-MIBG uptake in the LV myocardium, most notably in the posteroseptal basal segments.^9^ We, too, found a significantly lower LV/M ratio in our ARVC/D group, as compared to the reference cohort (3.2 vs 3.9; *P* = 0.014).

Previous studies demonstrated that a present sympathetic dysfunction, as visualized by ^123^I-MIBG imaging, predicts poor prognosis in heart failure patients.^6^,^15^ Paul et al. confirmed the generalizability of this relationship to ARVC/D without present heart failure.^10^ These patients had a significantly higher risk to develop life-threatening ventricular tachyarrhythmia to follow-up. Thus, there is an emerging role of ^123^I-MIBG SPECT/CT for individual risk stratification and the possibility to detect the disease in the early phase. However, we contend that previous methods have been inadequate since they were restricted to consideration of LV, and provided no information regarding the RV sympathetic innervation.^10^ We now used the superior spatial and anatomic assignment of SPECT/CT to assess separately the RV, which showed a significantly reduced RV/M ratio in the ARVC/D group as compared to the reference group (1.6 ± 0.3 vs. 2.0 ± 0.2; *P* = 0.001), and indeed proved to be the best method to distinguish between the two patient groups. Nonetheless, to establish the absolute magnitude of reduced RV innervation a comparison to individuals without heart disease will be mandatory.

It is well known that ARVC/D is characterized by a fibrofatty infiltration of the heart muscle, which primarily affects the right ventricle^1^,^20^ and in the further course of the disease can lead to the involvement of the left ventricle.^2^ During disease progression ^123^I-MIBG uptake is hampered due to the damaging of sympathetic fibers.^10^ This might also lead to a change in the patient’s RV/M, LV/M, and H/M ratios over the course of time, which could be a potential tool to non-invasively monitor disease progression.

### ROC-Analysis

ROC-analysis revealed a threshold for the H/M ratio of (1.66) comparable to that previously published for patients with heart failure (1.60) by Jacobson et al.,^15^ but with a rather low sensitivity (75%) and specificity (60%). The AUC of only 0.73 also indicates that planar H/M ratio is not adequate to distinguish the ARVC/D and IVF groups. In contrast, the SPECT/CT findings for the LV/M ratio showed clearly superior results for the cut-off threshold of 3.41, which gave higher sensitivity (77%), specificity (80%), and AUC (0.78). The best parameter of all was the RV/M ratio with a cut-off value of 1.88, which gave 94% sensitivity, 80% specificity, and an AUC of 0.93.

In this context, it is of great interest to compare the sensitivities and specificities of our SPECT/CT results with those of the modified task force major criteria; parameters determined by echocardiography required 95% specificity, with sensitivity of the individual parameters ranging from 55% to 75%. Based on our findings yielding a high specificity and sensitivity for the RV/M ratio the inclusion of the ^123^I-MIBG imaging in the revised McKenna criteria could be a future consideration. However, in order to establish this parameter for the diagnosis of ARVC/D, it would be essential to validate our findings in a larger cohort.

In addition, the progressive fibro-fatty replacement of the RV with a subsequent enlargement of the ventricle leads to abnormalities in the regional wall motion. These changes can be assessed by cardiac magnetic resonance imaging.^21^ Complementary nuclear medicine techniques like gated SPECT equilibrium radionuclide angiocardiography can be used to calculate ventricular functions, volumes, synchrony, and entropy or regional wall motion abnormalities. This techniques could be helpful in identifying patients with suspected ARVC/D^22^–^24^ and should be further evaluated in the future to enable a faster and more reliable diagnosis in this rare but endangered cohort.

### Limitations

A major limitation of our study is the low number of patients and the heterogeneous clinical stage of the disease, both of which are due to rarity of ARVC/D. Furthermore, the lack of a healthy control group—due to the ethical requirements of radiation exposure to healthy subjects—is limiting the transfer of this results into the clinical routine, since the published thresholds were established to distinguish the cardiac ^123^I-MIBG uptake between individuals with heart disease.

## Conclusion

ARVC/D can be diagnosed reliably based on structural abnormalities of the RV, but definitive diagnosis is difficult at early disease stages. The present SPECT/CT method allows for non-invasive evaluation of cardiac sympathetic function, with separate evaluation of left and right ventricular ^123^I-MIBG uptake. We found significantly reduced ^123^I-MIBG accumulation in the RV of ARVC/D patients in comparison to our reference group, such that the RV/M ratio confirmed diagnosis of ARVC/D with a high sensitivity and specificity. Validation of this finding in a larger cohort could be helpful in the management of patients with suspected ARVC/D. Future studies might also investigate cohorts with other electrophysiological cardiac disorders, e.g., right ventricular outlet tract tachyarrhythmias that currently pose major diagnostic dilemmas.

## New Knowledge Gained

Right ventricular cardiac ^123^I-MIBG uptake can be assessed using SPECT/CT imaging and used to distinguish between patients with suspected and proven ARVC/D. This might be helpful in the management of patients with suspected ARVC/D.

## Electronic Supplementary Material

Below is the link to the electronic supplementary material.

Supplementary material 1 (DOCX 2071 kb)

Supplementary material 2 (PPTX 356 kb)

## Abbreviations

^123^I-MIBG: ^123^I-Metaiodobenzylguanidine
ARVC/D: Arrhythmogenic right ventricular cardiomyopathy/dysplasia
AUC: Area under the curve
IVF: Idiopathic ventricular fibrillation
LV: Left ventricle
ROC: Receiver-operating-characteristics
ROIs: Regions of interest
RV: Right ventricle
SPECT: Single-photon computer tomography
VOIs: Volumes of interest
