# Supplementary material for: Assessment of right ventricular sympathetic dysfunction in patients with arrhythmogenic right ventricular cardiomyopathy: An 123I-metaiodobenzylguanidine SPECT/CT study
Source: J Nucl Cardiol. 2018 Dec 17;27(6):2402–9. doi: 10.1007/s12350-018-01545-3 (PMC7749057; doi:10.1007/s12350-018-01545-3)
Supplement: Supplementary file 1 — Supplementary material 1 (DOCX 2071 kb) [file 12350_2018_1545_MOESM1_ESM.docx]

*Preliminary SPECT-image analysis*

After importing the SPECT/CT images, global volumes of interest (VOI) were manually drawn around the left and right ventricles in the CT scan and applied to the SPECT images, upon confirmation of correct image registration. The manually drawn entire heart VOIs included the myocardium and the myocardial cavity. Subsequently threshold ranges relative to the hottest voxel (upper threshold 100%, lower threshold 30-70%, 10% steps) were applied to the manually drawn global VOI (figure A1; green 30-100% VOI for left ventricle, yellow 30-100% VOI for the right ventricle), generating a series of final VOIs for the quantification of the myocardial tracer uptake. Areas of the heart vulnerable to spillover from the liver were manually excluded from the VOI. A cubic mediastinum VOI (33 x 33 x 33 mm) was placed in the upper mediastinum, guided by the morphological information of the CT scan (figure A1). The ^123^I-MIBG uptake in the left (LV/M) and right ventricle (RV/M) was evaluated separately, and scaled to the uptake in the mediastinum for all tested threshold-ranges.


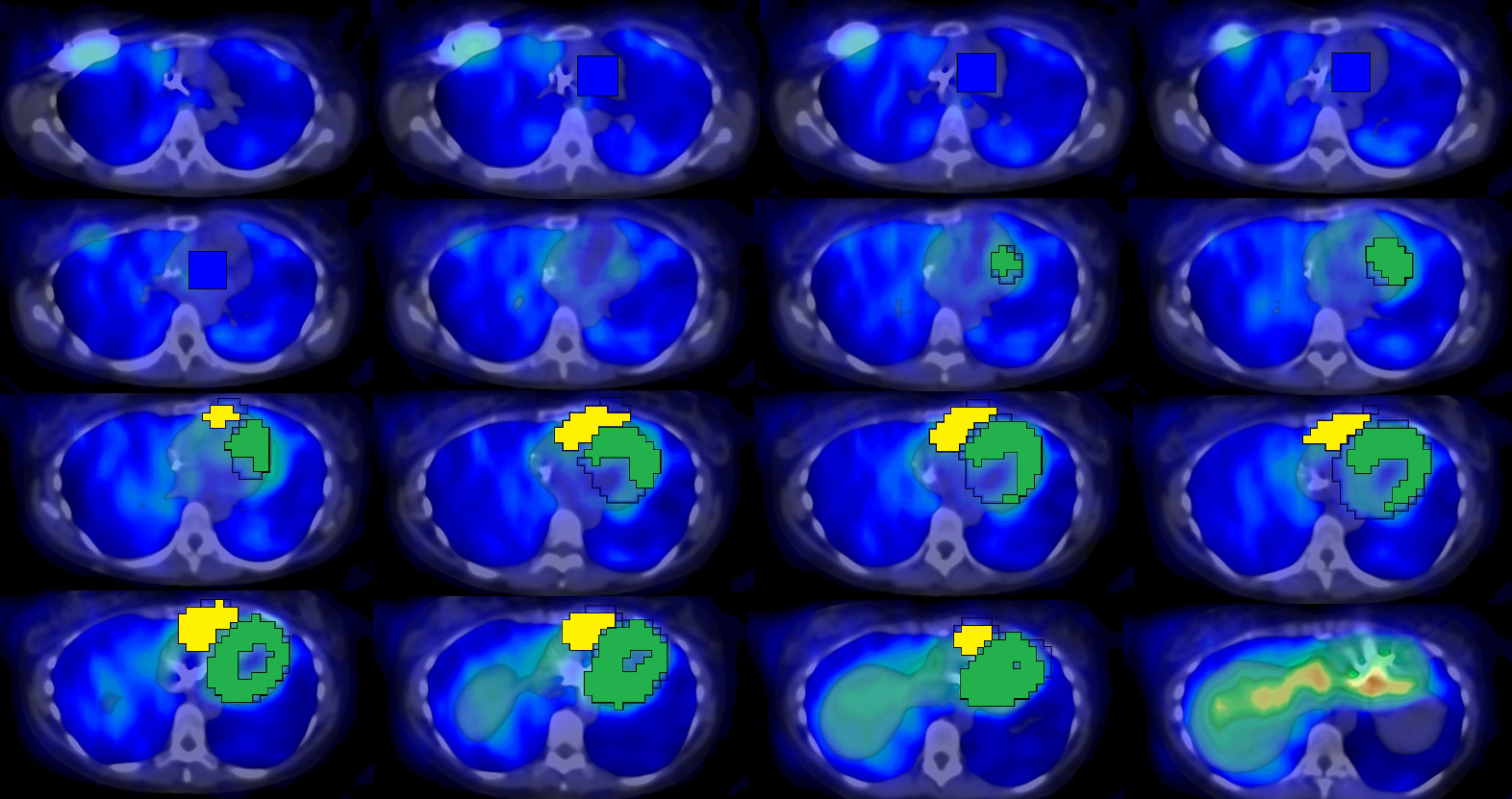


Figure A1: Hybrid SPECT/CT fusion scan in axial orientation from cranial (upper left) to caudal (lower right) slice. Representative images of the right (yellow) and left (green) ventricular VOIs (threshold 30-100%) as well as the mediastinum VOI (blue) for the quantification of myocardial MIBG uptake.

Means of ARVC/D and IVF group for each of the five tested threshold ranges were compared using the unpaired Student’s t-tests or the Mann-Whitney-U (groups without normal distribution). ROC analyses were performed for LV/M and RV/M ratios with each intensity threshold ranges, and the optimal cut-off value to differentiate the two clinical groups was identified from the sensitivity and specificity.

*Preliminary SPECT image analysis results*

Results are summarized for left ventricle (Table 1a) and right ventricle (Table 1b). The highest significance level for the LV/M ratio in correctly distinguishing the ARVC/D and IVF groups was found for the 70-100% threshold. Nonetheless, the other threshold ranges also gave a significant difference (p<0.05) in the LV/M ration between groups.

| **threshold** | **ARVC/D** | **IVF** | **P** |
| --- | --- | --- | --- |
| 30-100 % | 2.4 ± 0.4 | 2.8 ± 0.5 | 0.035 |
| 40-100 % | 2.6 ± 0.4 | 3.0 ± 0.6 | 0.035 |
| 50-100 % | 2.8 ± 0.5 | 3.3 ± 0.6 | 0.022 |
| 60-100 % | 3.0 ± 0.5 | 3.6 ± 0.7 | 0.019 |
| 70-100 % | 3.2 ± 0.5 | 3.9 ± 0.8 | 0.014 |

**Table 1a** Means ± SD und p-values for LV/M ratio

| **threshold** | **ARVC/D** | **IVF** | **P** |
| --- | --- | --- | --- |
| 30-100 % | 1.6 ± 0.3 | 2.0 ± 0.2 | 0.001 |
| 40-100 % | 1.7 ± 0.3 | 2.1 ± 0.3 | 0.001 |
| 50-100 % | 1.8 ± 0.3 | 2.2 ± 0.3 | 0.001 |
| 60-100 % | 1.9 ± 0.3 | 2.4 ± 0.4 | 0.001 |
| 70-100 % | 2.0 ± 0.3 | 2.5 ± 0.4 | 0.003 |

**Table 1b** Means ± SD und p-values for RV/M ratio

An ROC analysis was performed for LV/M and RV/M ratios for the five different threshold ranges, and the optimal cut-off value was estimated based on the highest sensitivity and specificity. The relevant results are summarized for LV/M (table 2a) and for RV/M (table 2b).

| **threshold** | **30-100 %** | **40-100 %** | **50-100 %** | **60-100%** | **70-100%** |
| --- | --- | --- | --- | --- | --- |
| cut-off | 2.48 | 2.68 | 2.90 | 3.08 | 3.17 |
| sensitivity | 65% | 65% | 65% | 65% | 48% |
| specificity | 80% | 80% | 80% | 80% | 90% |
| cut-off | 2.55 | 2.72 | 2.92 | 3.17 | 3.41 |
| sensitivity | 71% | 71% | 71% | 71% | 77% |
| specificity | 70% | 70% | 70% | 70% | 80% |
| cut-off | 2.62 | 2.81 | 3.15 | 3.44 | 3.84 |
| sensitivity | 77% | 77% | 82% | 82% | 88% |
| specificity | 60% | 60% | 60% | 60% | 50% |
| AUC | 0.74 | 0.75 | 0.76 | 0.77 | 0.78 |

**Table 2a** ROC analysis for LV/M with different threshold ranges

| **threshold** | **30-100 %** | **40-100 %** | **50-100 %** | **60-100%** | **70-100%** |
| --- | --- | --- | --- | --- | --- |
| cut-off | 1.80 | 1.88 | 1.96 | 2.04 | 2.13 |
| sensitivity | 82% | 77% | 82% | 82% | 71% |
| specificity | 100% | 100% | 100% | 100% | 100% |
| cut-off | 1.86 | 1.90 | 1.97 | 2.06 | 2.14 |
| sensitivity | 88% | 82% | 82% | 82% | 77% |
| specificity | 90% | 90% | 90% | 90% | 90% |
| cut-off | 1.88 | 1.96 | 2.03 | 2.13 | 2.22 |
| sensitivity | 94% | 94% | 88% | 88% | 88% |
| specificity | 80% | 80% | 80% | 80% | 80% |
| AUC | 0.93 | 0.92 | 0.92 | 0.90 | 0.88 |

**Table 2b** ROC analysis for RV/M with different threshold ranges

For the RV/M ratio, the optimal voxelwise intensity cut-off value was 1.86, which gave 88% sensitivity and 90% specificity of 90% using a threshold of 30-100%. Increasing the cut-off value to 1.88 would have increased the sensitivity at the expense of lesser specificity. The AUC at this threshold value was 0.93, and declined with increasing threshold-values to 0.88, as shown in table 2b.


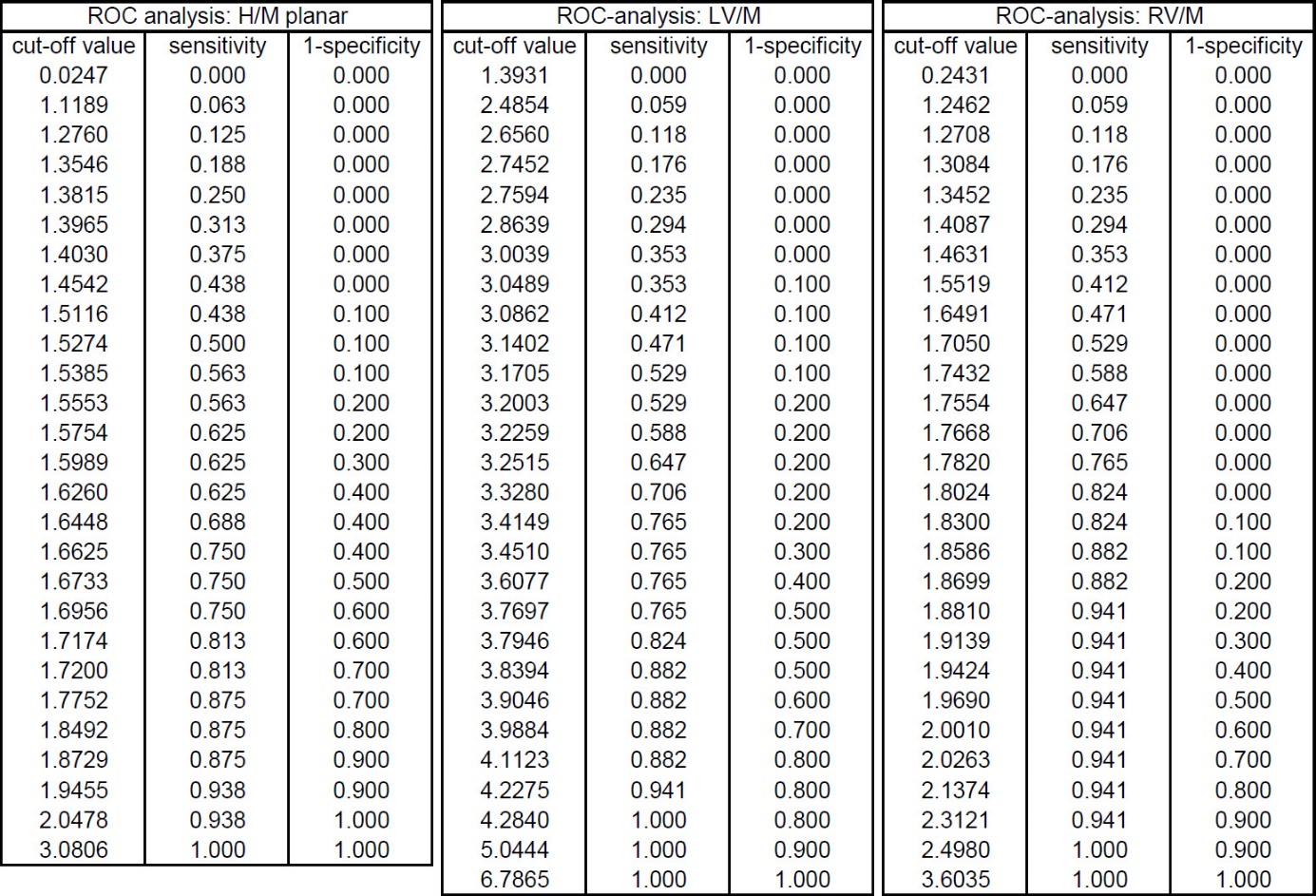


**Table 3:** Detailed ROC-analysis for H/M ratio planar, LV/M ratio (70-100%) and RV/M ratio (30-100%) (due to technical issues during the planar acquisition one patient hat to be excluded from the analysis)
