# Supplementary material for: Assessment of right ventricular sympathetic dysfunction in patients with arrhythmogenic right ventricular cardiomyopathy: An 123I-metaiodobenzylguanidine SPECT/CT study
Source: J Nucl Cardiol. 2018 Dec 17;27(6):2402–9. doi: 10.1007/s12350-018-01545-3 (PMC7749057; doi:10.1007/s12350-018-01545-3)
Supplement: Supplementary file 2 — Supplementary material 2 (PPTX 356 kb) [file 12350_2018_1545_MOESM2_ESM.pptx]

## Slide 1
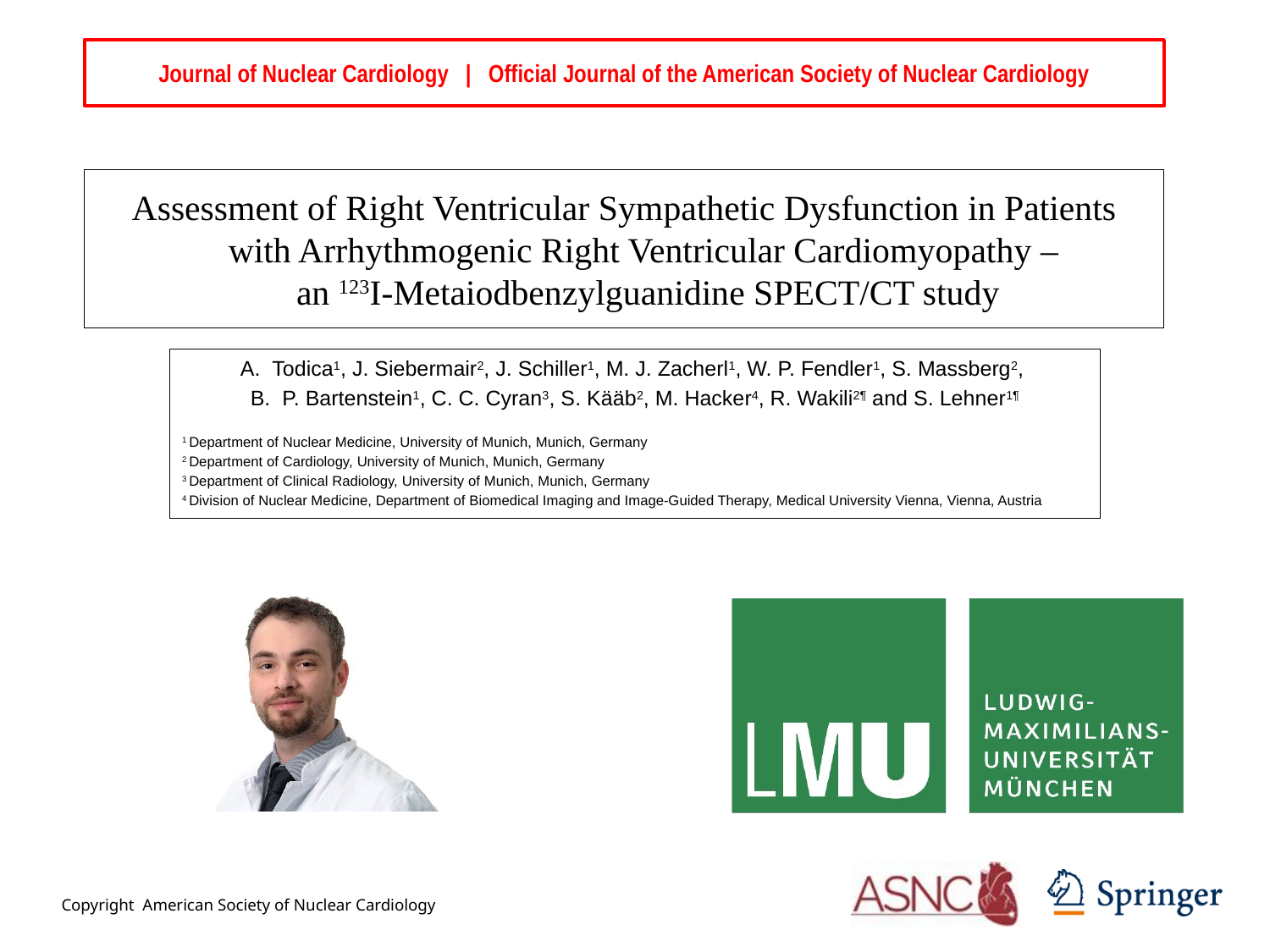

Journal of Nuclear Cardiology | Official Journal of the American Society of Nuclear Cardiology
# Assessment of Right Ventricular Sympathetic Dysfunction in Patients with Arrhythmogenic Right Ventricular Cardiomyopathy – an 123I-Metaiodbenzylguanidine SPECT/CT study
Todica1, J. Siebermair2, J. Schiller1, M. J. Zacherl1, W. P. Fendler1, S. Massberg2,
P. Bartenstein1, C. C. Cyran3, S. Kääb2, M. Hacker4, R. Wakili2¶ and S. Lehner1¶
1 Department of Nuclear Medicine, University of Munich, Munich, Germany
2 Department of Cardiology, University of Munich, Munich, Germany
3 Department of Clinical Radiology, University of Munich, Munich, Germany
4 Division of Nuclear Medicine, Department of Biomedical Imaging and Image-Guided Therapy, Medical University Vienna, Vienna, Austria
Copyright American Society of Nuclear Cardiology

## Slide 2
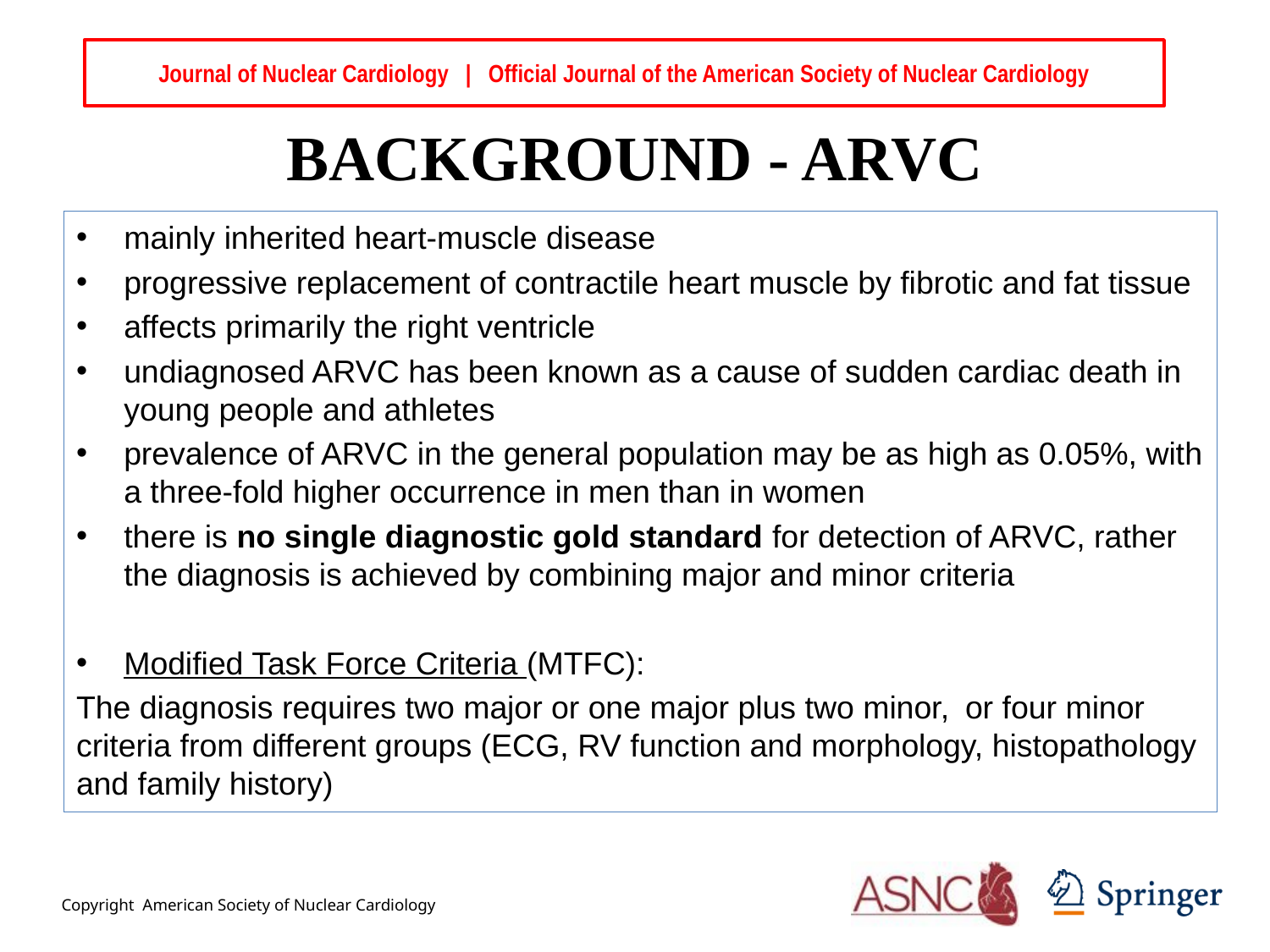

Journal of Nuclear Cardiology | Official Journal of the American Society of Nuclear Cardiology
# BACKGROUND - ARVC
mainly inherited heart-muscle disease
progressive replacement of contractile heart muscle by fibrotic and fat tissue
affects primarily the right ventricle
undiagnosed ARVC has been known as a cause of sudden cardiac death in young people and athletes
prevalence of ARVC in the general population may be as high as 0.05%, with a three-fold higher occurrence in men than in women
there is no single diagnostic gold standard for detection of ARVC, rather the diagnosis is achieved by combining major and minor criteria
Modified Task Force Criteria (MTFC):
The diagnosis requires two major or one major plus two minor, 	or four minor criteria from different groups (ECG, RV function and morphology, histopathology and family history)
Copyright American Society of Nuclear Cardiology

## Slide 3
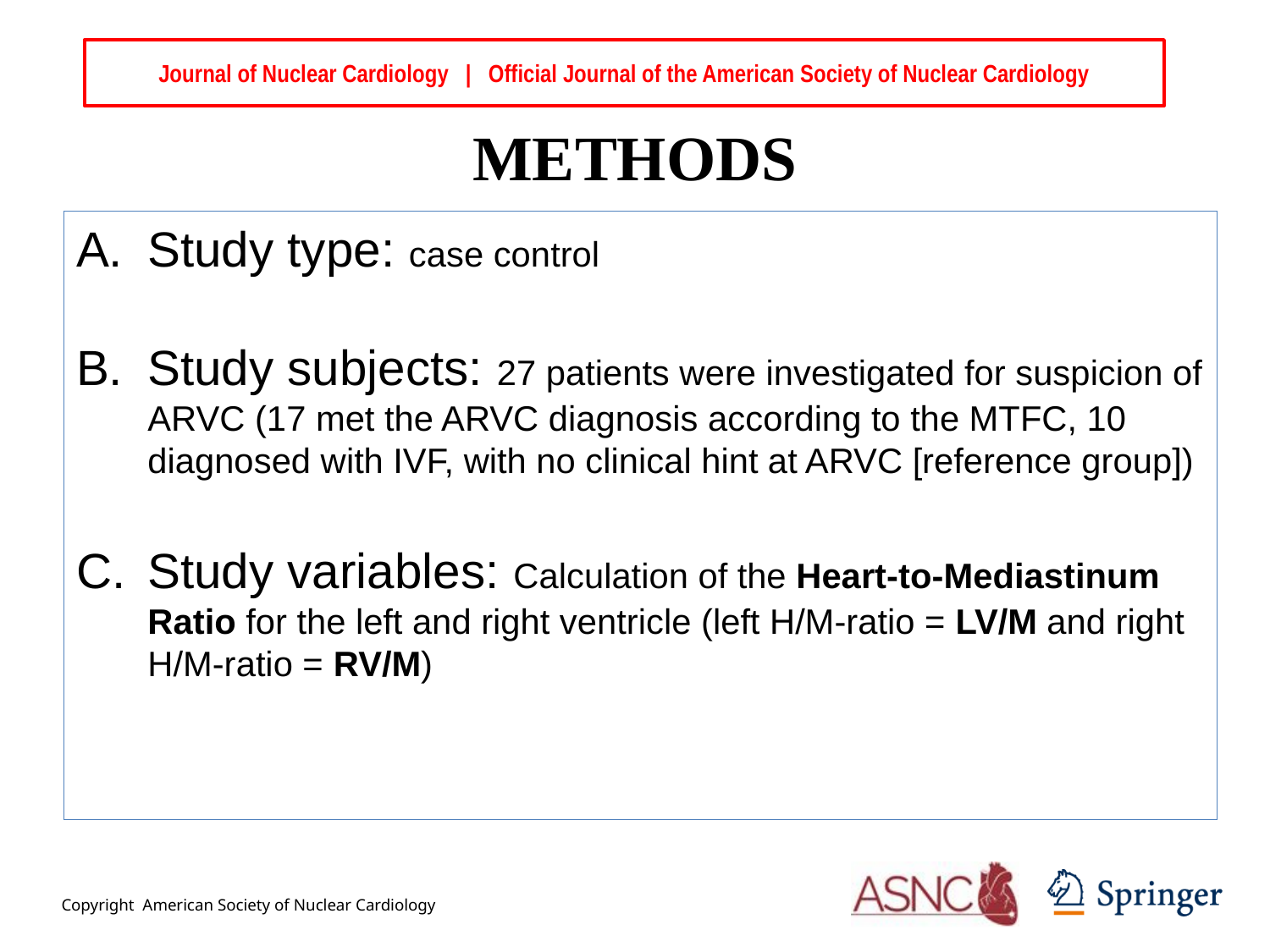

Journal of Nuclear Cardiology | Official Journal of the American Society of Nuclear Cardiology
# METHODS
Study type: case control
Study subjects: 27 patients were investigated for suspicion of ARVC (17 met the ARVC diagnosis according to the MTFC, 10 diagnosed with IVF, with no clinical hint at ARVC [reference group])
Study variables: Calculation of the Heart-to-Mediastinum Ratio for the left and right ventricle (left H/M-ratio = LV/M and right H/M-ratio = RV/M)
Copyright American Society of Nuclear Cardiology

## Slide 4
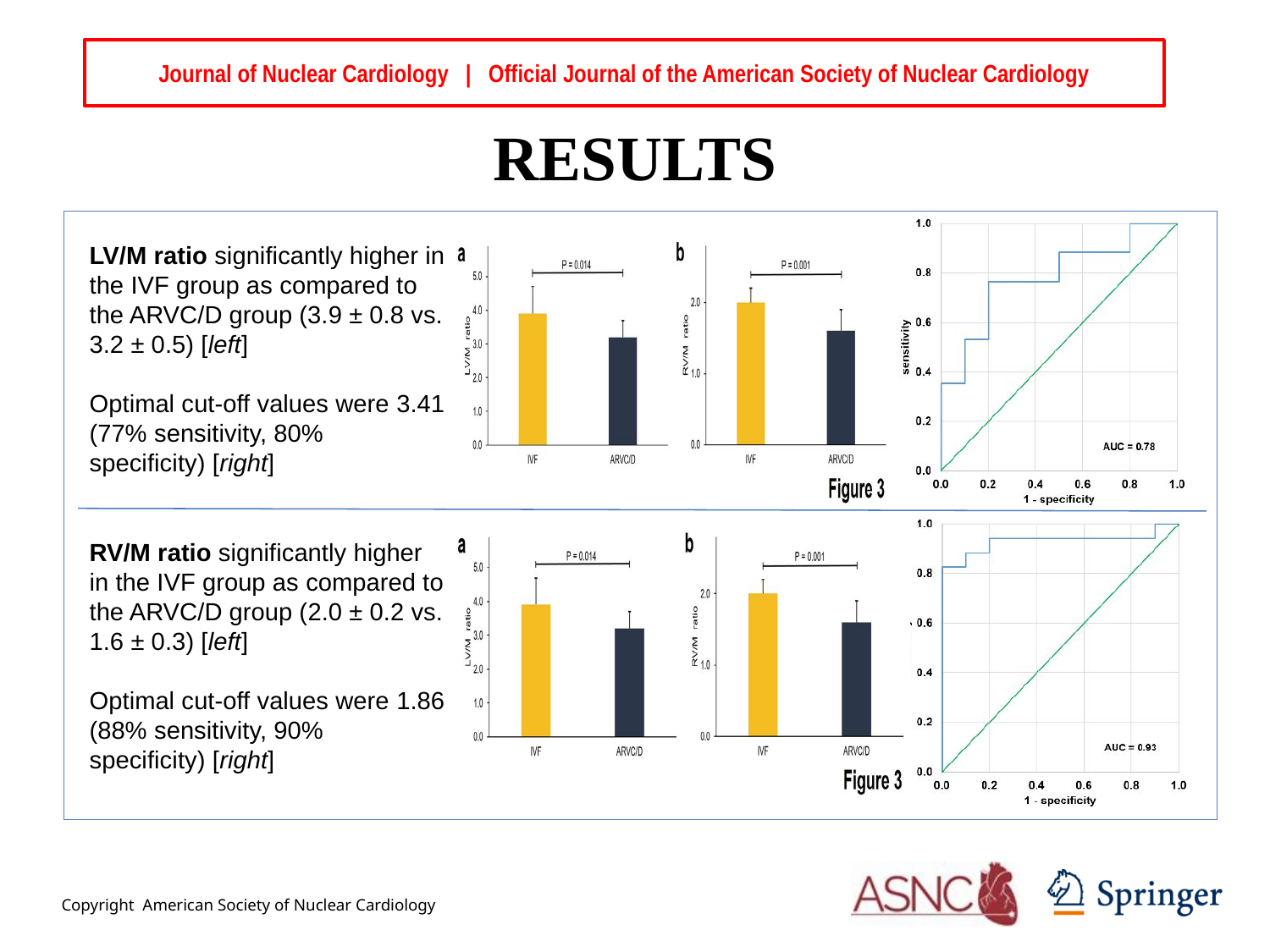

Journal of Nuclear Cardiology | Official Journal of the American Society of Nuclear Cardiology
# RESULTS
LV/M ratio significantly higher in the IVF group as compared to the ARVC/D group (3.9 ± 0.8 vs. 3.2 ± 0.5) [left]
Optimal cut-off values were 3.41 (77% sensitivity, 80% specificity) [right]
RV/M ratio significantly higher in the IVF group as compared to the ARVC/D group (2.0 ± 0.2 vs. 1.6 ± 0.3) [left]
Optimal cut-off values were 1.86 (88% sensitivity, 90% specificity) [right]
Copyright American Society of Nuclear Cardiology

## Slide 5
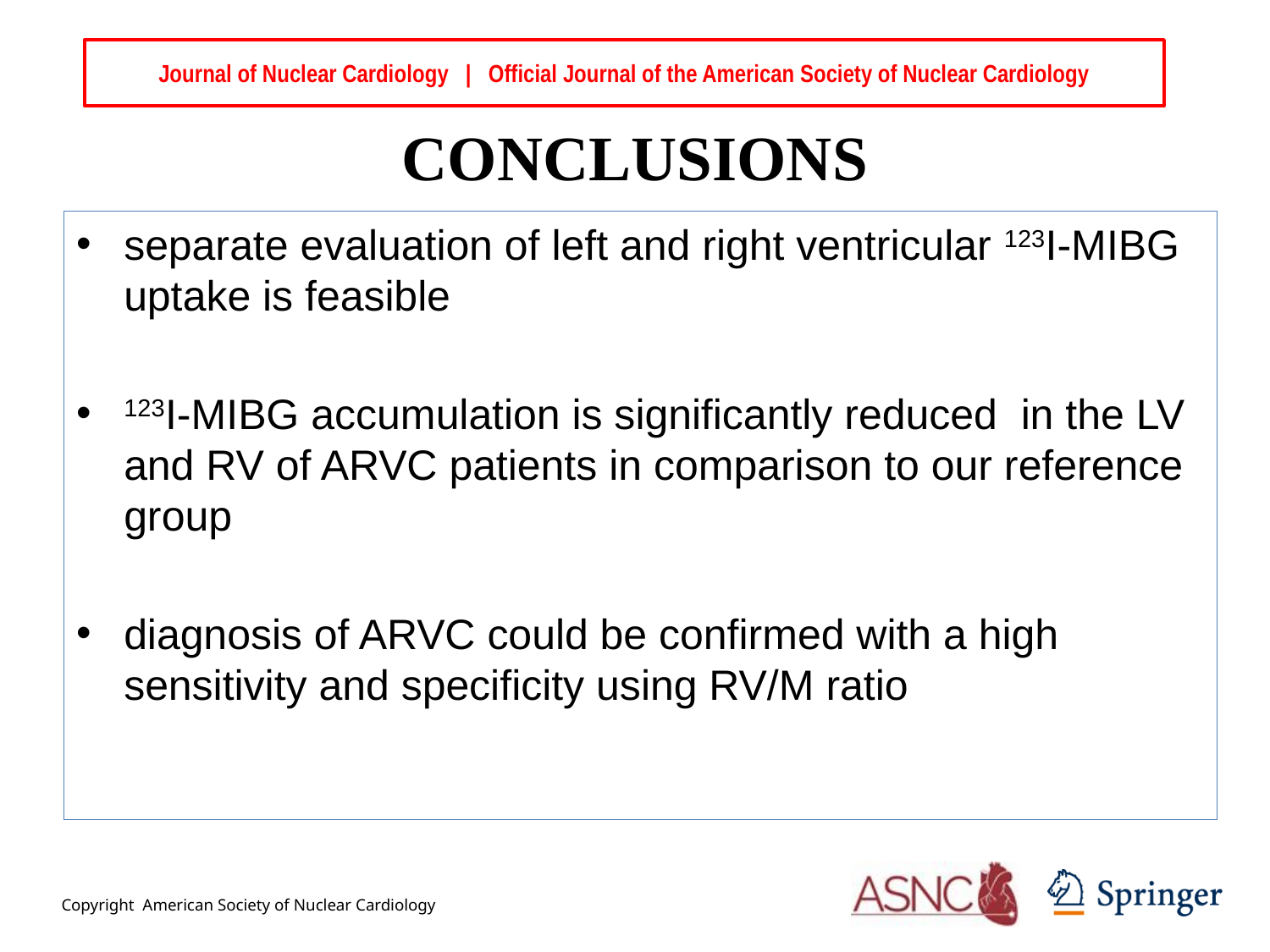

Journal of Nuclear Cardiology | Official Journal of the American Society of Nuclear Cardiology
# CONCLUSIONS
separate evaluation of left and right ventricular 123I-MIBG uptake is feasible
123I-MIBG accumulation is significantly reduced in the LV and RV of ARVC patients in comparison to our reference group
diagnosis of ARVC could be confirmed with a high sensitivity and specificity using RV/M ratio
Copyright American Society of Nuclear Cardiology
